# Human T-Cell Lymphotropic Virus Type 1 and *Strongyloides stercoralis* Co-infection: A Systematic Review and Meta-Analysis

**DOI:** 10.3389/fmed.2022.832430

**Published:** 2022-02-14

**Authors:** Lingqing Ye, Graham P. Taylor, Carolina Rosadas

**Affiliations:** ^1^Section of Virology, Department of Infectious Disease, Imperial College London, London, United Kingdom; ^2^National Centre for Human Retrovirology, St. Mary's Hospital, Imperial College Healthcare NHS Trust, London, United Kingdom

**Keywords:** HTLV-1, *Strongyloides stercoralis*, co-infections, severity, treatment, prevalence

## Abstract

**Background:**

The distribution of human T cell lymphotropic virus type 1 (HTLV-1) overlaps with that of *Strongyloides stercoralis*. *Strongyloides stercoralis* infection has been reported to be impacted by co-infection with HTLV-1. Disseminated strongyloidiasis and hyperinfection syndrome, which are commonly fatal, are observed in HTLV-1 co-infected patients. Reduced efficacy of anti-strongyloidiasis treatment in HTLV-1 carriers has been reported. The aim of this meta-analysis and systematic review is to better understand the association between HTLV-1 and *S. stercoralis* infection.

**Methods:**

PubMed, Embase, MEDLINE, Global Health, Healthcare Management Information Consortium databases were searched. Studies regarding the prevalence of *S. stercoralis*, those evaluating the frequency of mild or severe strongyloidiasis, and treatment response in people living with and without HTLV-1 infection were included. Data were extracted and odds ratios were calculated. Random-effect meta-analysis was used to assess the pooled OR and 95% confidence intervals.

**Results:**

Fourteen studies were included after full-text reviewing of which seven described the prevalence of *S. stercoralis* and HTLV-1. The odds of *S. stercoralis* infection were higher in HTLV-1 carriers when compared with HTLV-1 seronegative subjects (OR 3.2 95%CI 1.7–6.2). A strong association was found between severe strongyloidiasis and HTLV-1 infection (OR 59.9, 95%CI 18.1–198). Co-infection with HTLV-1 was associated with a higher rate of strongyloidiasis treatment failure (OR 5.05, 95%CI 2.5–10.1).

**Conclusion:**

*Strongyloides stercoralis* infection is more prevalent in people living with HTLV-1. Co-infected patients are more likely to develop severe presentation and to fail treatment. Screening for HTLV-1 and *Strongyloides* sp. should be routine when either is diagnosed.

## Introduction

Human T-cell lymphotropic virus type 1 (HTLV-1) is a retrovirus that belongs to the *Deltaretrovirus* genus in the *Retroviridae* family (1). This enveloped, single-stranded positive sense RNA virus discovered in 1979, is the first reported human retrovirus. HTLV-1 infection is identified worldwide, especially in subtropical and tropical countries, such as Southwest Japan, West Africa, South America, and the Caribbean. An estimated 5–10 million individuals are infected with HTLV-1, of which 90–95% are asymptomatic. This may be an underestimate as many regions have not reported the prevalence of HTLV-1 (2). HTLV-1 infects mainly CD4+ T lymphocytes and causes life-long infection.

Asymptomatic infection can progress to severe, or even fatal diseases. Adult T-cell leukemia/lymphoma (ATL/ATLL) with a median survival <1 year, develops in 5% HTLV-1 infected patients (3, 4). HTLV-1-associated myelopathy / tropical spastic paraparesis (HAM/TSP), a progressive inflammation in the central nervous system (CNS) (5), occurs in ~3% (6, 7) whilst a range of other inflammatory conditions (8), such as uveitis (9), Sjogren's syndrome (10), and bronchiolitis (11) along with several cancers may be associated with HTLV-1 infection. Importantly, HTLV-1 infection is associated with an unexplained 57% increase in overall mortality (12). This could be partly explained by the impact of HTLV-1 on co-infections.

*Strongyloides stercoralis* has two life cycles, a free-living cycle in soil and a parasitic cycle in the host (mainly human). In the free-living cycle, eggs are hatched as rhabditiform larvae, transformed into infectious filariform larvae, which penetrate the skin and migrate to the intestine where they mature into adult females and produce eggs parthenogenetically. The eggs hatch as non-infective rhabditiform larvae and are excreted in feces. In the soil or feces rhabditiform larvae are fertilized to filariform larvae. However, autoinfection also occurs in which the rhabditiform larvae fertilize into filariform larvae in the large bowel, migrate to the lungs and return to the bowel through swallowed sputum, *allowing S. stercoralis* to persist (13, 14).

Although ~50% patients with strongyloidiasis are asymptomatic, hyperinfection syndrome (HS) and disseminated strongyloidiasis (DS) are severe clinical presentations with 85–100% mortality (15–17). HS is the uncontrolled proliferation of larvae during immune compromise and characterized by a high number of larvae in sputum and stool. In DS larvae are detected in organs other than associated with the autoinfection cycle (lung and intestine). High strongyloidiasis treatment failure rate has been reported in HTLV-1 positive patients.

Here, we conducted a systematic review and meta-analysis of the association between HTLV-1 and strongyloidiasis infections, the impact of HTLV-1 co-infection on strongyloidiasis severity, and on anti-helminth treatment efficacy.

## Methods

### Search Strategy and Study Selection

This systematic review and meta-analysis were performed following the guideline of PRISMA (Preferred Reporting Items for Systematic Reviews and Meta-analysis). PubMed and OVID (MEDLINE, Embase, Healthcare Management Information Consortium, Global Health) were used as the electronic databases (up to 7 January, 2021). Keywords used in the search were “*Strongyloides stercoralis*,” “*S. stercoralis*,” “Strongyloides,” “Strongyloidiasis,” “Human T-lymphotropic virus,” “Human T-cell Leukemia Virus,” “Human T-cell Leukemia Virus,” “HTLV-1,” and “HTLV”. Studies were included if they focused on at least one of these areas: (1) the prevalence of *Strongyloides stercoralis* and HTLV-1 in the population; (2) the severity of strongyloidiasis in people living with HTLV-1 and HTLV-1 uninfected individuals; (3) the efficacy of anti-helminthic treatment in *S. stercoralis*-infected patients co-infected with HTLV-1 or without HTLV-1. Duplicate studies, review articles and articles: that were not written in English, Portuguese, Spanish or Chinese; where full text was not available; or had not been peer reviewed, were excluded. Studies without a control group and those without confirmatory test for HTLV-1 infection (Western Blot, Line Immunoassay and/or PCR), those focusing only on changes in biomarkers, for example, eosinophils count and IgE levels, and HTLV-1 proviral load, were also excluded. Severe strongyloidiasis was defined as hyperinfection, dissemination and the identification of *S. stercoralis*-larvae in specimen other than stool sample as well as the definition used by the authors.

### Data Extraction and Analysis

For each paper that met the selection criteria, the following data were extracted when available: year of publication, locations, regions and country of the study, total number of participants, number of positive subjects (infected by HTLV-1 and/or Strongyloides), methods for *S. stercoralis* and HTLV-1 diagnosis. To analyze the clinical outcome, the number of patients with severe strongyloidiasis and those without in case and control groups was extracted. For studies relating to efficacy of treatment, the number of patients with evidence of treatment failure, the dosages and the types of drugs used were collected.

Meta-analysis was performed using software Revman5. A random-effect model was used to calculate the odds ratio of *Strongyloides stercoralis* infection among the HTLV-1 positive and HTLV-1 negative populations and 95% confidence intervals (CI). The OR of *S. stercoralis* infection among people living with HTLV-1 (PLHTLV) when different diagnostic methods were used was calculated in the subgroup analysis (stool analysis or serological techniques). A forest plot for this subgroup analysis was plotted. Odds ratio (OR) and 95% CI were calculated for the effect of HTLV-1 on *S. stercoralis* infection, i.e., severity and treatment results. Heterogeneity in all meta-analyses was calculated. The statistical significance level considered was *p* < 0.05.

## Results

**Figure 1:**
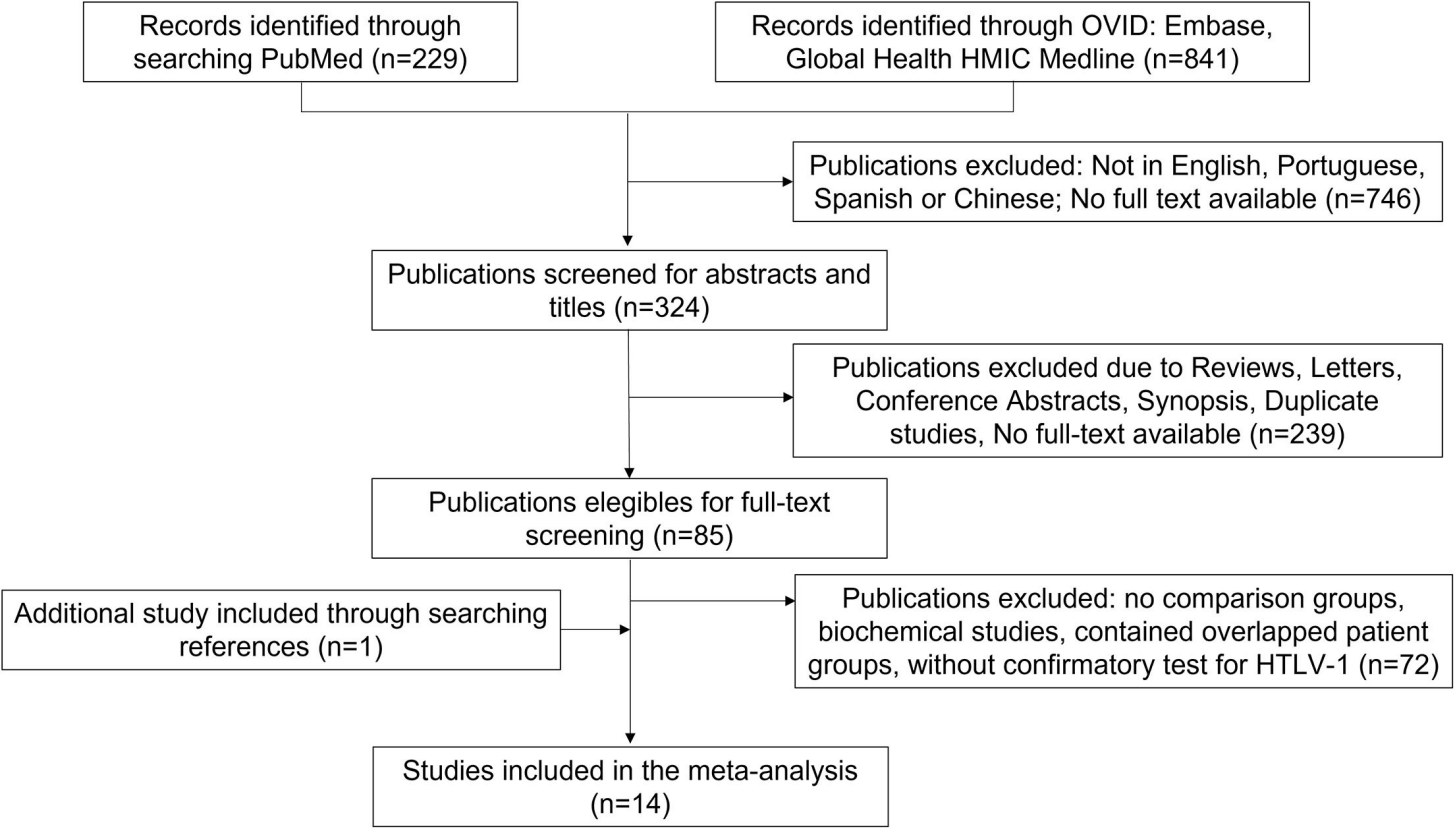
Flow chart of the selection of studies.

One thousand hundred and seventy studies had been retrieved according to the keywords. Three hundred and twenty-four studies were full-text available articles and written in English, Portuguese, Spanish or Chinese. Eighty-five papers were screened using full-text and 14 studies that met the inclusion criteria were used in the meta-analysis (Figure 1).

### The Risk of *Strongyloids* sp. Infection Is Higher in People Living With HTLV-1

Seven studies examined the prevalence of HTLV-1 and *Strongyloides stercoralis* co-infection: two from Australia (18, 19), one from French West Indies (20), two from Brazil (21, 22), two from Jamaica (23, 24). Both studies from Brazil used stool examination for the diagnosis of *S. stercoralis* (21, 22). Serological examination was used in four studies (18–20, 24) and one study from Jamaica used both types of examination (23) (Table 1).

**Table 1:** Studies reporting the prevalence of *S. stercoralis* and HTLV-1 that met the selection criteria.

**References**	**Country**	**Number of participants**	**H+SS+ ***n*** (%)**	**H+SS- ***n*** (%)**	**H-SS+ ***n*** (%)**	**H-SS- ***n*** (%)**	**Diagnosis of HTLV-1**	**Diagnosis of SS**
Einsiedel et al. (18)	Australia	72	6 (8.3)	22 (30.6)	4 (5.6)	40 (55.6)	EIA, PA, WB, PCR	Serology
Robinson et al. (23)	Jamaica	207	14 (6.8)	9 (4.3)	48 (23.2)	136 (65.7)	ELISA, WB	Serology
			10	4	7	41	ELISA, WB	Stool
Chieffi et al. (19)	São Paulo, Brazil	152	11 (7.2)	80 (52.6)	1 (0.7)	60 (39.5)	ELISA, WB	Stool
Einsiedel et al. (19)*	Australia	950	111 (9.9)	237 (26.5)	158 (13.9)	444 (49.6)	PA, EIA, IFA, WB	Serology
Chaturvedi et al. (24)	Jamaica	288	17 (5.9)	117 (40.6)	18 (6.3)	136 (47.2)	EIA, WB, PCR	Serology
Furtado et al. (22)	Pará, Brazil	100**	6 (6.0)	36 (36.0)	3 (3.0)	55 (55.0)	ELISA, PCR	Stool
Courouble et al. (20)	Guadeloupe, French West Indies	238	37 (15.5)	82 (5.5)	13 (5.5)	106 (44.5)	EIA, WB	Serology

*H, Human T-cell lymphotropic Virus Type 1; SS, Strongyloides stercoralis. + and – present positive and negative results. ELISA, Enzyme-linked immunosorbent assay; WB, Western blot; PA, Particle agglutination; EIA, Enzyme immunoassay; IFA, Immunofluorescence assay; PCR, Polymerase chain reaction; NR, Not reported*.

* *Patients with borderline Strongyloids serology were not included*.

** *Patients infected with HTLV-2 and their relatives, were included in the group not infected by HTLV-1*.

Two studies from Brazil compared the frequency of larvae positive stool samples in patients with HTLV-1 and a control group. Chieffi et al. (21) evaluated blood donors from São Paulo and observed that the frequency of Ss infection was higher in those infected by HTLV-1 when compared to the control group [11/91 (12.1%) vs. 1/61 (1.6%), OR = 8.25 (1.05–175.6), *p* = 0.015]. In Pará, the detection of Ss larvae was more frequent in patients with HTLV-1 than in their relatives, not infected by this virus [6/42 (14.3%) vs. 0/29 (0%), *p* = 0.036] (22). In the same study the frequency of Ss in HTLV-2 patients and their relatives was also assessed but no significant difference was observed [2/18 (11.1%) vs. 1/11 (9.1%), *p* = 0.684] (22).

Four studies compared the prevalence of antibodies to Ss in patients with HTLV-1 and a control group. One study from Jamaica, found similar prevalence of antibodies to Ss in blood donor candidates with and without HTLV-1 infection [HTLV-1 seropositive: 17/134 (12.7%) vs. HTLV-1 seronegative: 18/154 (11.7%), *p* = 0.79] (24). In a remote indigenous community from Australia, although the prevalence of Ss antibodies was higher in those infected by HTLV, the difference was not statistically significant [6/28 (21.4%) vs. 4/44 (9.1%), *p* = 0.17] (18). The same research group then evaluated a higher number of participants and the results whilst similar approaching statistical significance [111/409 (27.1%) PLHTLV-1 had positive Strongyloides serology compared with 158/717 (22%) of those who were not infected with HTLV-1, *p* = 0.063] (19). In Guadeloupe, French West Indies, blood donors seropositive for HTLV-1 had 3.68 (95% CI 1.74–7.89) higher frequency of having antibodies to Ss than HTLV-1 uninfected blood donors [37/119 (31.1%) vs. 13/119 (10.9%)]. When adjusting for age, the risk was two times higher for people living with HTLV-1 (AdjOR = 2.08, 95%CI 1–4.35) (20).

**Figure 2:**
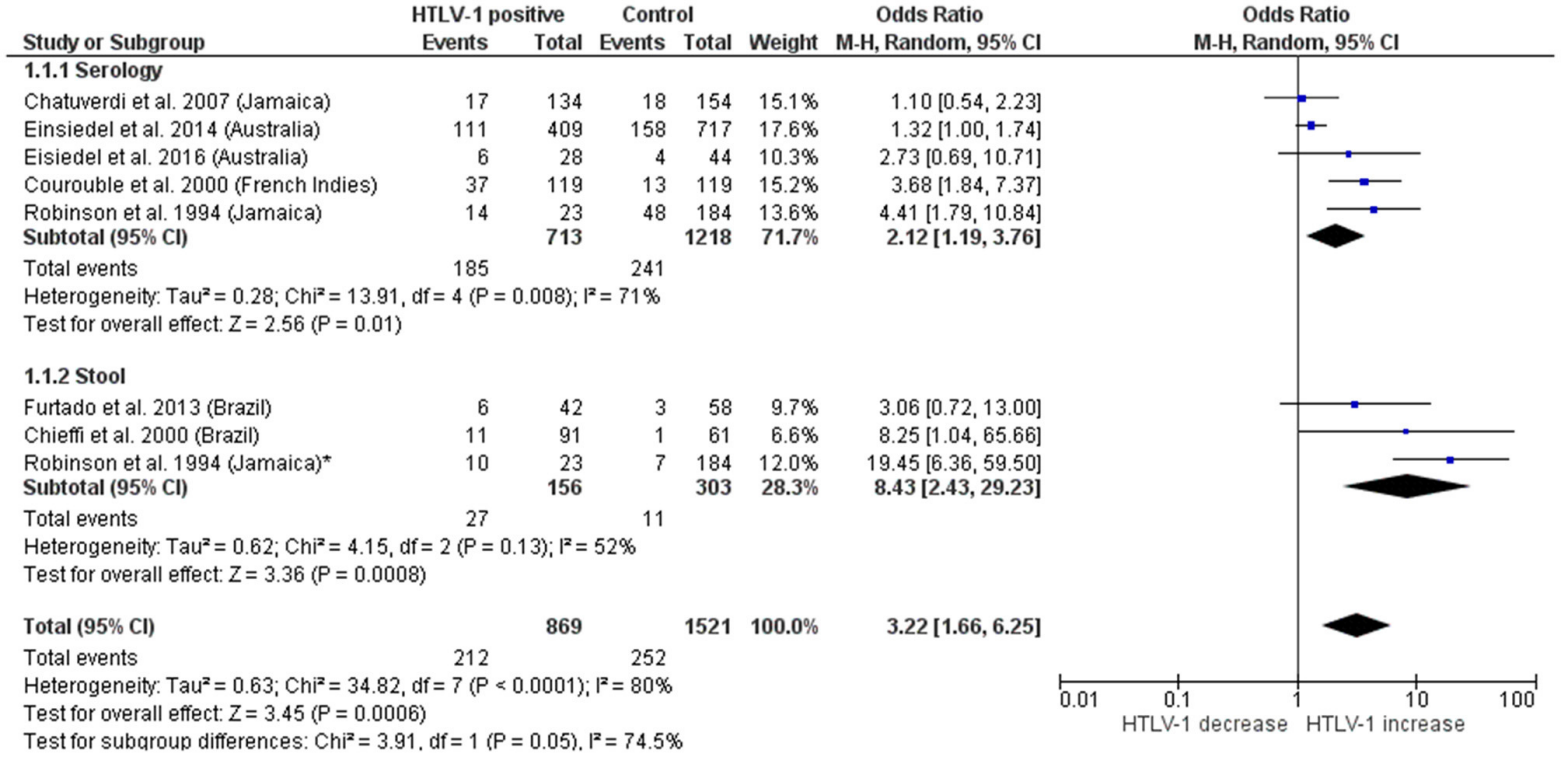
Forest plot of subgroup meta-analysis of the association between *S. stercoralis* and HTLV-1 using different diagnostic methods.

Another study from Jamaica found evidence of an association between *Strongyloides* and HTLV-1 infection (Table 1). In that occasion, 17 patients had been diagnosed positive for *S. stercoralis*-larvae of whom 10 (58.8%) were co-infected with HTLV-1. In contrast, 4 of 45 (8.9%) individuals with *S. stercoralis*-seropositive but larva negative were HTLV-1 positive and only 9 out of 145 (6.2%) participants not infected by *S. stercoralis* (evaluated by serology and stool tests) were HTLV-1 positive. This study gave evidence that being a *S. stercoralis* carrier was highly associated with the presence of HTLV-1 (OR 19.45, 95% CI 6.36–59.5; Figure 2) (23).

In total, 2,390 participants were included (869 individuals infected with HTLV-1 and 1,521 seronegative for HTLV-1) (Figure 2). *Strongyloides stercoralis* infection was more frequently observed in patients with HTLV-1 24.4% (212/869) compared with 16.6% in HTLV-1 uninfected individuals (252/1,521), with a pooled odds ratio 3.22 [95% Confidence interval (95%CI) = 1.66–6.25]. However, large heterogeneity was observed between different studies (*I*^2^ = 63%, *P* = 0.001). The association was stronger when analyzing patients diagnosed by stool methods [OR (95%CI) = 8.43 (2.4–29.2)] than those diagnosed with serological examination [OR (95%CI) = 2.12 (1.2–3.8)]. A high heterogeneity was observed within subgroups and among the studies that used serology (Figure 2).

### Patients With HTLV-1 Are More Likely to Develop Severe Strongyloidiasis

**Figure 3:**
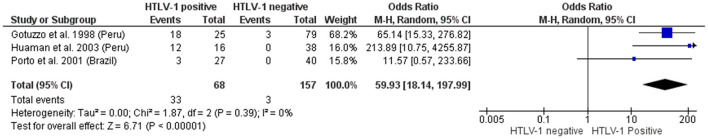
Meta-analysis of the association between severe strongyloidiasis and HTLV-1.

Three studies reporting the association between the severity of strongyloidiasis, and HTLV-1 co-infection met the inclusion criteria (Figure 3) (25–27).

One study found all Peruvian patients with Strongyloidiasis hyperinfection to be HTLV-1 positive (12/12). On the other hand, only 4 of 42 (9.5%) patients with asymptomatic or mild strongyloidiasis had concurrent HTLV-1 (0/12 asymptomatic, 4/30 symptomatic). This gives the odds of severe strongyloidiasis to be 214 times (10.7–4,256) higher among PLHTLV compared to those without HTLV-1 infection, although the 95% CI was broad (27). In another study from Peru, the great majority of patients with severe strongyloidiasis were co-infected by HTLV-1 (18/21, 85.7%). The frequency of HTLV-1 infection was significantly higher in patients with severe disease comparing to those with intestinal Ss (6/62; 9.7%, *p* < 0.001) and to age-matched healthy individuals with negative stool samples for Ss (1/21, 4.7%) (26). The group reported that two patients with intestinal Ss co-infected by HTLV-1 eventually evolved to hyperinfection despite two courses of ivermectin treatment (26). In both studies from Peru, severe cases were defined by “systemic illness (chronic diarrhea, abdominal pain, loss of weight, cough, edema, hypoproteinemia, and anemia), and with two or more organs (usually lung, intestines, liver, and the central nervous system) involved, with stools positive for *Strongyloides* larvae, and at least one larvae in a sputum specimen” (26, 27). Severe strongyloidiasis was not found among HTLV-1 negative subjects from Brazil (0/40). In contrast, 3/27 of PLHTLV had severe strongyloidiasis, characterized by hospitalization due to severe Ss symptoms (diarrhea, dehydration and hypoalbuminemia) (OR = 11.57, 95%CI 0.6–233.7) (Figure 3) (25). Interestingly, all three patients did not have anti-Ss IgE in serum nor a reactive skin test to Ss, despite being severely ill with anti-Ss IgG in serum and positive stool samples (25).

Furtado et al. (22) reported that 2/6 patients with HTLV-1 and Ss co-infection had hyperinfection, but no case of Ss infection was reported in the control group, uninfected by HTLV-1, and therefore this study was not included in this analysis.

In total, 33/68 patients with HTLV-1 and Strongyloids had severe strongyloidiasis, while only 3/157 individuals without HTLV-1 co-infection had severe presentation of Ss infection, resulting in a OR = 59.9 (18.1–198).

### HTLV-1 Infection Negatively Impacts Treatment Response to *S. stercoralis*

**Figure 4:**
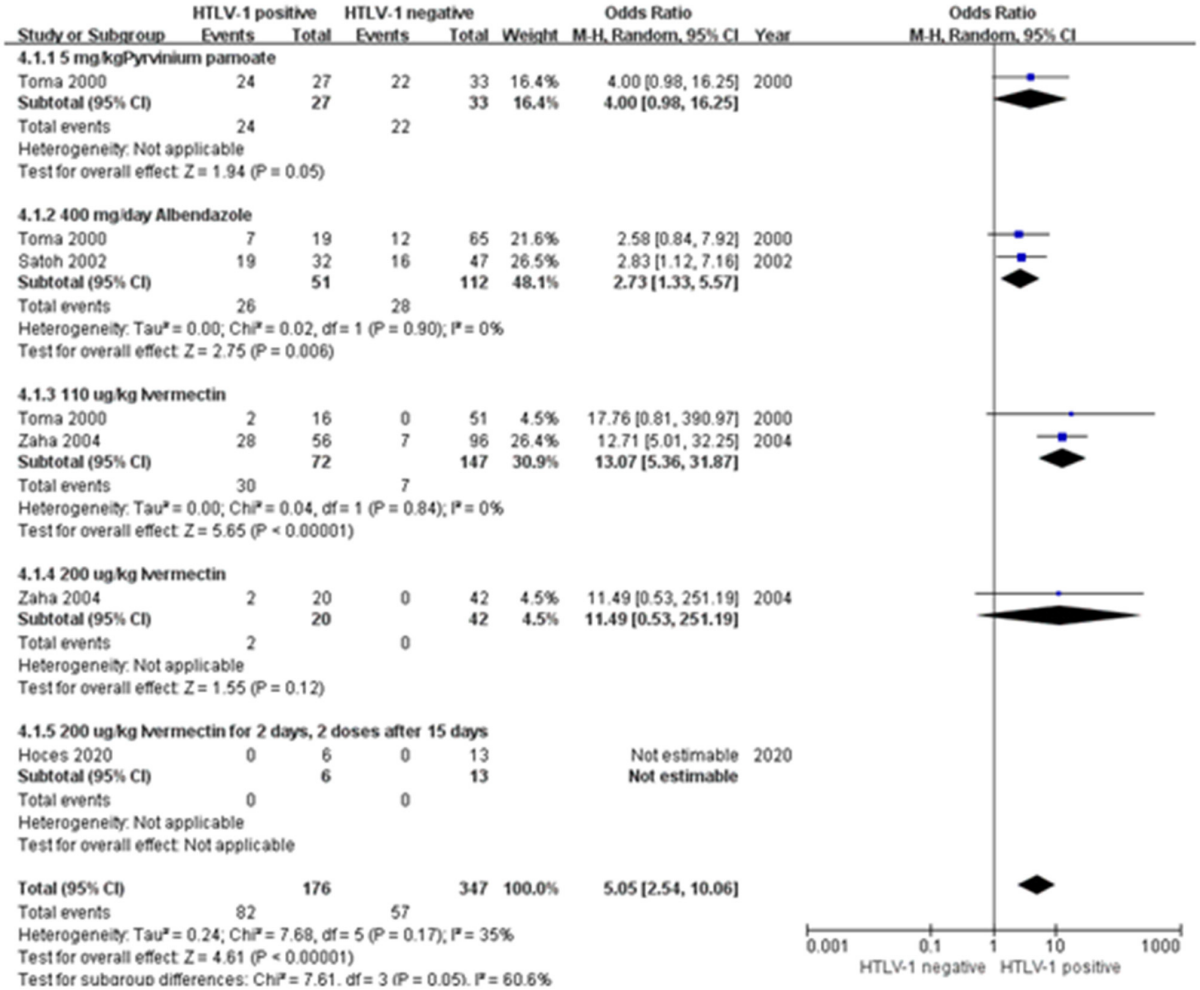
Forest plot of meta-analysis on treatment failure according to HTLV-1 serostatus.

When considering the impact of HTLV-1 on anti-*S. stercoralis* treatment response, three studies from Japan and one study from Peru found association between treatment failure and HTLV-1 infection (Figure 4) (28–31). Data were presented according to the drugs used in the studies (Table 2). The pooled odds ratio of anthelminthic treatment failure among HTLV-1 positive was 5 times higher than among HTLV-1 negative individuals (95% CI: 2.54–10.06) (Figure 4). Most studies assessed the efficacy at 12-month, varying from 3 to 12 months. All treatment regimens comprised two courses with a 2-week interval before the second course was taken. Toma et al. observed that the cure rates were significantly lower in PLHTLV-1 regardless the treatment used (30). The highest failure rate was observed when using Pyrvinium pamoate, with 24/27 (88.9%) HTLV-1 seropositive and 22/33 (66.7%) HTLV-1 seronegative patients failing to eradicate the larvae (*p* < 0.05) (30). Response to Albendazole (400 mg/day) was also lower in co-infected patients (36.8% of HTLV-1 positive patients failed treatment compared to 18.5% monoinfected, *p* < 0.02). Even when treated with Ivermectin (1 tablet, 6 mg/tablet, ~110 μg/kg), which was considered the most effective drug among the three analyzed in this study, patients co-infected with HTLV-1 failed treatment more frequently than those seronegative for HTLV-1 [2/16 (12.5%) vs. 0/51(0%), *p* < 0.002] (30). Zaha et al. (31) compared the anthelmintic effect of two different doses of ivermectin (110 and 200 μg/Kg) in patients with and without HTLV-1 coinfection. Although he found a significant increase in eradication of *S. stercoralis* larvae when treated with the higher dosage of Ivermectin, the cure rate was consistently lower in those PLHTLV. The long-term (4–12 months) anthelminthic effect of 110 μg/kg Ivermectin in HTLV-1 co-infected patients was 50% (28/56) and significantly increased to 90% (18/20, *p* < 0.05), when the dosage increased to 200 μg/kg (31). The increased dosage also increased the efficacy of treatment in patients with strongyloidiasis only. None of the 42 HTLV-1 negative patients who received 200 μg/kg Ivermectin failed the treatment compared to 7/96 when using the lower dose (31). Hoces et al. found that all patients with strongyloidiasis responded to two-course treatment with 200 μg/kg Ivermectin followed by 400 μg/kg Ivermectin 15 days later, independent of HTLV-1 serostatus (28).

**Table 2:** Summary of treatment efficacy of strongyloidiasis in patients with or without HTLV-1 according to the type of drugs used in the treatment.

**References**	**Country**	**Follow-up duration**	**Treatment**	**HTLV-1 positive**		**HTLV-1 negative**	
				**Success**	**Failure**	**Success**	**Failure**
Zaha et al. (31)	Japan	12 months	6 mg (110 μg/kg) Ivermectin, repeated after 2 weeks	28	28	89	7
			200 μg/kg Ivermectin, repeated after 2 weeks	18	2	42	0
Satoh et al. (29)	Japan	12 months	400 mg/day Albendazole for 3 days, repeated after 2 weeks	13	19	31	16
Toma et al. (30)	Japan	12 months	5 mg/kg Pyrvinium pamoate for 3 days, repeated after 2 weeks	3	24	11	22
			400 mg/day Albendazole for 3 days, repeated after 2 weeks	12	7	53	12
			1 tablet Ivermectin (6 mg), repeated after 2 weeks	14	2	51	0
Hoces et al. (28)	Peru	3–6 months	200 μg/kg Ivermectin for 2 days, 2 × 200 μg/kg Ivermectin after 15 days	6	0	13	0

A subgroup meta-analysis on the association of treatment failure and HTLV-1 that used different drugs was performed. The pooled OR for the non-cured cases in the patients co-infected with HTLV-1 vs. without HTLV-1 treated with 400 mg/day Albendazole was 2.73 (95% CI 1.33–5.57) and for those treated with Ivermectin 110 μg/kg (1 tablet, 6 mg) was 13.07 (95% CI 5.36–31.87). In total the OR of treatment failure was 5.05 (95% CI = 2.5–10.1). The heterogeneity between subgroups was large and reaching statistical significance (*I*^2^ = 60.6%, *p* = 0.05) (Figure 4).

## Discussion

A 2013 systematic review found an association between the prevalence of *Strongyloides* and HTLV-1 infection (32) whilst Schierhout et al. reported a strong association between HS and HTLV-1 infection (12). Here, the impact of HTLV-1 infection on the prevalence of *S. stercoralis* when diagnosed by either serology or microscopy, on the severity of disease and anthelminthic treatment efficacy have been systematically reviewed.

In total, 14 studies from both *S. stercoralis* and HTLV-1 endemic areas were included in this meta-analysis, of which five were from South America, four from Japan, three from the Caribbean and two from Australia. Compared with uninfected persons the risk of *S. stercoralis* infection is increased (OR 3.22) amongst HTLV-1 infected persons. This extends the earlier observations of Schär et al. (32) (OR 2.48, 95% CI 0.70–9.03) although their results did not reach statistical significance. As expected, the prevalence of *S. stercoralis* infection, in HTLV-1 infected persons, when determined by serology (22.1%) is higher than that determined by stool examinations (10%). However, the odds for being HTLV-1 seropositive are higher when Ss is detected in stool (OR 8.43), compared to those positive for *S. stercoralis* by serology (OR 2.12). This suggests that persistent parasite excretion contributes to these associated prevalence rates, rather than incidence of infection. However, it remains possible that patients infected with HTLV-1 are at higher risk of becoming infected with *S. stercoralis*, although this may be due to co-factors such as socio-economic and environmental characteristics. The heterogeneity across the studies that used serological tests was large and significant (*I*^2^ = 71%, *p* < 0.05), while studies using stool examinations were more homogenous (*I*^2^ = 52%, *p* = 0.13). Although the findings are robust, the number of studies remain limited and further investigation from a wider range of countries, using robust diagnostics test and an adequate number of participants are required to better understand the contribution of each (risk of infection or parasite persistence) to the overall association.

The present study confirmed not only that *Strongyloides* infection is more commonly observed in PLHTLV, but in addition, that HTLV-1 infection is more frequently associated with a severe outcome. The present study demonstrated that the chance of developing severe strongyloidiasis is almost 60 times higher if the person is coinfected with HTLV-1 (OR = 59.9, 95%CI 18.1–199). The number of manuscripts that matched the inclusion criteria is small and were restricted to two South American countries (Peru and Brazil). However, there are enough biological data to support this finding. Experimental data suggest that HTLV-1, unlike HIV, activates, rather than kills, CD4+ T cells which upregulates Interleukin (IL)-2/IL-2 receptor and subsequently induce Th1 responses (33, 34). In contrast, this causes a reduction in the Th2 response which is important for the eradication of the parasite. A suppressed immune response against helminths, characterized by low level of eosinophils, IgE, IL-5, and IL-4, was found in people co-infected with HTLV-1 and *S. stercoralis* (25, 35, 36). Recent studies suggest that regulatory T cells are expanded in patients living with HTLV-1, which blunts the Th2 responses. Furthermore, steroids and other immunomodulatory drugs used as routine treatment for HTLV-1 associated diseases, may contribute to increase the risk of severe disease (37).

In addition to the evidences showing plausible immunological basis underlying the association between HTLV-1 infection and increased disease severity in Ss co-infected patients, Einsiedel et al. (19) observed that HTLV-1 carriers in Central Australia were more likely to be admitted in the hospital with a diagnosis of Strongyloidiasis and had higher admission numbers due to this condition [admission rates HTLV-1 positive (*n* = 490) = 0.23 vs. HTLV-1 negative (*n* = 827) = 0.11, *p* < 0.0001]; Adjusted negative binominal regression coefficient = 0.563, *p* = 0.005). This epidemiological data also supports the findings of our study.

Another concern that needs attention is that the impact of this co-infection is not restricted to unfavorable outcome of Ss, but it may also negatively impact the outcome of HTLV-1 infection. A number of studies reported higher HTLV-1 proviral load among co-infected individuals, a known risk factor for the development of HTLV-1 associated disease (36, 38, 39). In addition, Ss stimulates oligoclonal proliferation of HTLV-1-infected lymphocytes and, in patients with this coinfection, HTLV-1 clonality is less stable over time, which may be a precipitating factor for ATL (39, 40). In fact, the latency of onset of ATL was shortened in patients with *S. stercoralis* (36, 40). Higher rates of mother-to-child HTLV-1 transmission was also reported among those women that were co-infected by Ss (41).

Altogether, these findings point to a negative impact of *S. stercoralis* infection in the outcome of HTLV-1 infection and the interplay between Ss and HTLV-1 in the pathogenesis of both infections was recently reviewed. Nevertheless, the authors highlighted that in the absence of suitable animal models, the mechanisms in disrupting host's immune system between HTLV-1 and *S. stercoralis* is poorly studied (42).

Treatment efficacy of different anti-*S. stercoralis* drugs in patients with or without HTLV-1 infection was also assessed. Efficacy varied by treatment with some being very poor and perhaps now obsolete. Nevertheless, regardless of treatment type, HTLV-1 co-infected patients had higher odds of having failed antiparasitic therapy than those without HTLV-1. The effective of treatments were determined by stool examinations with a post-treatment follow-up duration ranging from 3 to 12 months, with a PCR test used in a proportion of patients in one study. It is important to undertaken proper follow-up of patients under/post-treatment and to perform multiple samplings to avoid false-negative stool examination results caused by the intermittent excretion of larvae (43, 44). All treatment regimens included in the analysis comprised two courses with a 2-week interval, taking into consideration drug toxicity and *S. stercoralis* autoinfection cycle, which takes about 3 or 4 weeks for eggs fertilizing to larvae and these to settle into the small bowel (44). The most widely used and most effective agent was Ivermectin. Ivermectin, approved since 2000 was the most efficient drug to eradicate *S. stercoralis*, especially in immunocompromised patients (45). Hoces et al. reported that all patients that had been treated with higher dosages ivermectin showed negative results, after treatment. However, late recurrences, even after two courses of appropriately doses ivermectin may occur, months or years later. In HTLV-1 co-infection, prolonged surveillance post-*Strongyloides* treatment is essential. Also, prompt treatment of strongyloidiasis in patients with HTLV-1 is important, as it resulted in increased TNFα levels and decreased the risk of recurrence (46). This shows the importance of diagnosing either HTLV-1 or *S. stercoralis*. Whatsoever, as discussed above, there is not such a “golden standard” of diagnosis of *S. stercoralis*, which poses difficulties for routine diagnosis. As both *S. stercoralis* and HTLV-1 may cause chronic and asymptomatic infection, active surveillance on those patients who are diagnosed with these pathogens is important. In addition, co-infection cases may also occur in non-prevalent areas due to influx of immigrants or prior travel to endemic countries.

## Conclusion

People living with HTLV-1 have a higher risk of being infected by *Strongyloides* and have higher risk of developing severe strongyloidiasis and anthelmintic treatment failure. Patients who have been infected with *S. stercoralis*, especially those who had traveled to or live in an endemic area, should be advised to be tested for HTLV-1. HTLV-1 patients who live in a *S. stercoralis* endemic area should be tested for *S. stercoralis*, especially before starting treatment for HTLV-1 associated diseases, in order to prevent the development of hyperinfection and disseminated strongyloidiasis. All patients who had developed a HS or DS should be screened for HTLV-1 as well as those who failed treatment to strongyloidiasis. Increasing awareness about the impact of this coinfection among general population and healthcare workers is needed, as well as further studies about the impact of HTLV-1 in other co-infections.

## Data Availability Statement

The original contributions presented in the study are included in the article/supplementary material, further inquiries can be directed to the corresponding author.

## Author Contributions

LY conducted the literature search, undertook the analyses, and wrote the first draft. CR and GPT designed and supervised the work and edited the final version. All authors contributed to the article and approved the submitted version.

## Funding

GPT was supported by Imperial NIHR Biomedical Research Center.

## Conflict of Interest

The authors declare that the research was conducted in the absence of any commercial or financial relationships that could be construed as a potential conflict of interest.

## Publisher's Note

All claims expressed in this article are solely those of the authors and do not necessarily represent those of their affiliated organizations, or those of the publisher, the editors and the reviewers. Any product that may be evaluated in this article, or claim that may be made by its manufacturer, is not guaranteed or endorsed by the publisher.
